# Loss of *Hoxa5* function affects *Hox* gene expression in different biological contexts

**DOI:** 10.1038/s41598-024-81867-0

**Published:** 2024-12-28

**Authors:** Béatrice Frenette, Josselin Guéno, Nicolas Houde, Kim Landry-Truchon, Anthony Giguère, Theyjasvi Ashok, Abigail Ryckman, Brian R. Morton, Jennifer H. Mansfield, Lucie Jeannotte

**Affiliations:** 1 Centre de Recherche sur le Cancer de L’Université Laval, Centre de Recherche du CHU de Québec-Université Laval (Oncology), 1401, 18e Rue, Québec, QC G1J 1Z4 Canada; 2 Department of Molecular Biology, Medical Biochemistry and Pathology, Université Laval, Québec, Canada; 3 Department of Biology, Barnard College, Columbia University, 3009 Broadway, New York, NY 10027 USA

**Keywords:** *Hoxa5*, *Hox* expression, *Hoxa5* mutant mice, Chromatin landscape, Respiratory system, Musculoskeletal system, Developmental biology, Molecular biology

## Abstract

*Hoxa5* plays numerous roles in development, but its downstream molecular effects are mostly unknown. We applied bulk RNA-seq assays to characterize the transcriptional impact of the loss of *Hoxa5* gene function in seven different biological contexts, including developing respiratory and musculoskeletal tissues that present phenotypes in *Hoxa5* mouse mutants. This global analysis revealed few common transcriptional changes, suggesting that HOXA5 acts mainly via the regulation of context-specific effectors. However, *Hox* genes themselves appeared as potentially conserved targets of HOXA5 across tissues. Notably, a trend toward reduced expression of *HoxA* genes was observed in *Hoxa5* null mutants in several tissue contexts. Comparative analysis of epigenetic marks along the *HoxA* cluster in lung tissue from two different *Hoxa5* mutant mouse lines revealed limited effect of either mutation indicating that *Hoxa5* gene targeting did not significantly perturb the chromatin landscape of the surrounding *HoxA* cluster. Combined with the shared impact of the two *Hoxa5* mutant alleles on phenotype and *Hox* expression, these data argue against the contribution of local *cis* effects to *Hoxa5* mutant phenotypes and support the notion that the HOXA5 protein acts in *trans* in the control of *Hox* gene expression.

## Introduction

*Hox* genes encode an evolutionary conserved family of transcription factors that play central regulatory roles in body patterning and development^1–3^. In mammals, 39 *Hox* genes are tightly organized in four clusters, *HoxA* to *HoxD* located on different chromosomes. This arrangement is fundamental for the transcriptional regulation and thus function of each gene. Initiation of *Hox* transcription is coordinated, with spatio-temporal activation occurring in sequence from 3ʹ to 5ʹ across each cluster. As a result, anterior to posterior regions of the body express distinct combinations of *Hox* genes, and HOX proteins act coordinately to confer positional identity. Establishment and maintenance of *Hox* expression include regulatory mechanisms that operate at the level of individual genes as well as other shared across several genes or whole clusters^4^.

The requirements for *Hox* genes have been extensively studied through molecular genetic analyses. *Hox* mutant mice display a panoply of phenotypes from skeletal transformations to organ defects and postnatal anomalies, indicating the broad range of action of *Hox* genes throughout life^3^. Mis-regulation of *Hox* gene expression is related to various pathologies including both positive and negative actions on tumor formation and metastasis^5^. Mutations in *Hox* genes are also associated with various human disorders^6^.

Despite their central developmental roles, there remains a paucity of information regarding downstream HOX-dependent regulatory networks. Defining HOX transcriptional targets *in vivo* and how HOX proteins achieve specificity is challenging, due in part to functional redundancy among *Hox* genes as well as their pleiotropy, overlapping expression domains, and similar DNA sequence recognition by HOX proteins. Whole genome transcriptomic, chromatin accessibility and chromatin interaction studies have shed light on HOX activities and targets in various cellular contexts^7–10^. An emerging view is that the tissue-dependence of *Hox* activity, which is apparent in phenotypic outcomes, results from regulation of cell-dependent target genes and genetic networks^11–13^. However, few studies have explicitly compared a *Hox* mutant’s transcriptional effect across different tissue types.

*Hoxa5* is a perfect paradigm for addressing the mechanistic basis of HOX protein cell-specificity due to its well characterized, non-redundant roles across a spectrum of tissues from the respiratory, musculoskeletal, digestive, reproductive, and nervous systems^14^. Indeed, *Hoxa5* is unusual among *Hox* genes because it is required for viability: most *Hoxa5* mutants die at birth from respiratory failure, and surviving mutants have respiratory deficiencies^15,16^. *Hoxa5* is required for proper diaphragm innervation and musculature, lung and trachea patterning and cell type specification, and post-natal alveolar development and function^17–21^. The extensive scope and the severity of these phenotypes establish the functional predominance of *Hoxa5* in respiratory tract morphogenesis. In the musculoskeletal system, *Hoxa5* mutants present homeotic transformations in the cervico–thoracic region of the vertebral column, patterning defects in the pectoral girdle and sternum, and changes in skeletal muscle and brown adipose tissue (BAT) depot size^15,22–24^. Altogether, the unusual range and severity of *Hoxa5* phenotypes relative to other single *Hox* mutants reveals a prevalent function for *Hoxa5* at this axial domain.

Although the diverse roles of *Hoxa5* in development are well established, little is known about the gene networks it regulates in any context. To examine the molecular impact of the *Hoxa5* loss of function, we performed RNA sequencing (RNA-seq) assays in selected respiratory and musculoskeletal tissues and timepoints. Overall, conserved target genes across contexts were rare. However, we observed a trend toward a broad *Hox* gene mis-regulation, suggesting that *Hox* genes may be common targets of HOXA5 across tissues. To exclude the possibility of *cis*-acting effects of the genome modifications used to disrupt *Hoxa5*, we performed a comparative analysis of *Hox* expression and epigenetic marks along the *HoxA* cluster in *Hoxa5* mutant mouse lines generated with two different targeting strategies^15,25^. The results, combined with the shared defects associated with the two *Hoxa5* mutant alleles, argue against the possibility that *cis*-acting effects contribute to *Hoxa5* mutant phenotypes and support the notion that HOXA5 protein participates in *trans* in the control of *Hox* gene expression.

## Results

### Bulk RNA-seq reveals few common HOXA5 gene targets

To explore the transcriptome-wide changes resulting from the loss of *Hoxa5* gene function across space and time, we applied bulk RNA-seq to tissues from the developing respiratory and musculoskeletal systems that present critical phenotypes in *Hoxa5* mouse mutants^21,23^. Tissue samples were collected from wild-type (wt) and *Hoxa5*^-/-^ null embryos. They included: trachea and lung, which come from the outgrowth of the foregut endoderm; diaphragm with its dual origins comprising muscle progenitors from cervical (C) somites C3 to C5 and the lateral plate-derived muscle connective tissue; interscapular BAT (iBAT), which arises at the brachial and cervical levels from the dermomyotome of somites; and somites from the C3 to the second thoracic (T2) axial domain, which contain precursors of musculoskeletal tissues, diaphragm and BAT^26–28^. Trachea, lung and diaphragm were collected at embryonic day (E) 15.5, when *Hoxa5* is highly expressed, for comparison of its roles between respiratory tissues. iBAT was sampled at E18.5, after activation of differentiation markers of brown adipocytes. To assess the transcriptomic effects of *Hoxa5* at different timepoints, two stages were analyzed for lung, E12.5 and E15.5, and somites, E10.5 and E12.5.

**Fig. 1:**
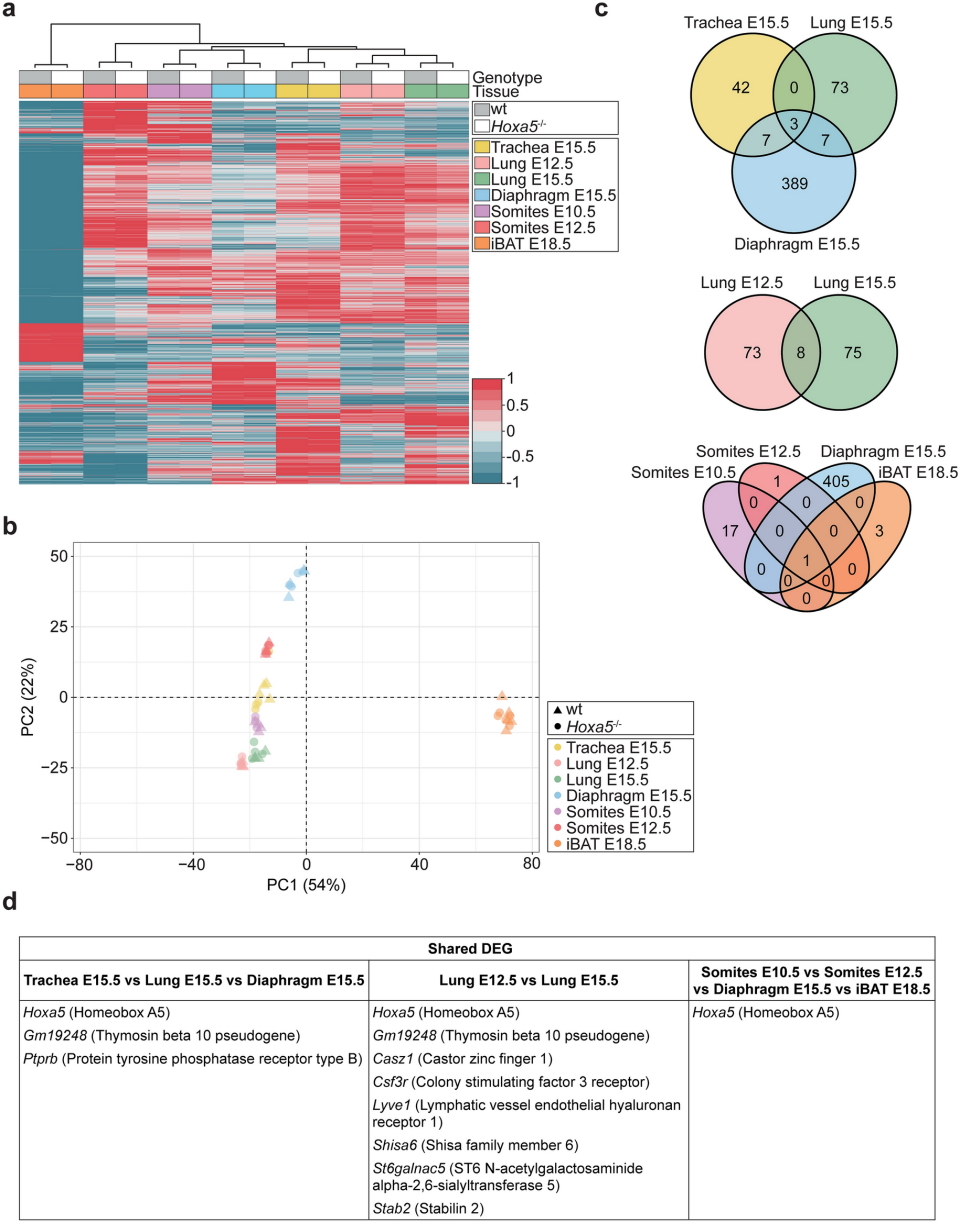
Overview of differential gene expression in *Hoxa5*^-/-^ mutant tissues. (**a**) Heatmap and hierarchical clustering based on comparison of gene expression in wt (grey) and *Hoxa5*^-/-^ (white) samples across the seven biological conditions tested represented by specific color: trachea at E15.5 (yellow), lung at E12.5 (pink), lung at E15.5 (green), diaphragm at E15.5 (blue), somites at E10.5 (purple), somites at E12.5 (red) and iBAT at E18.5 (orange). Data are represented in z-score of the Log_2_ of the mean of TPM + 1. Genes with a mean of TPM < 1 in all conditions and genotypes were excluded from the analyses. (**b**) Principal Component Analysis (PCA) of wt (triangle) and *Hoxa5*^-/-^ (circle) specimens for trachea at E15.5 (yellow), lung at E12.5 (pink), lung at E15.5 (green), diaphragm at E15.5 (blue), somites at E10.5 (purple), somites at E12.5 (red) and iBAT at E18.5 (orange). (**c**) Venn diagrams comparing differentially expressed genes (DEG) in different tissue contexts. Differential expression analysis was performed with DESeq2 with an *alpha* parameter of 0.05. Statistically significant differential expression was defined as FC ≥ 1.5 and padj < 0.05 between wt and *Hoxa5*^-/-^ conditions. (**d**) Lists of the shared DEG depicted in C.

All expressed genes were used to perform hierarchical clustering of samples. Heatmap analysis showed clustering of specimens according to tissue rather than genotype (Fig. 1a). Clustering patterns largely followed developmental relationship of tissues: trachea and lung specimens were grouped together, while diaphragm and somites at E10.5 clustered jointly. Somites at E12.5 followed by iBAT at E18.5 presented the most differences with the other tissues. This distribution was also observed following Principal Component Analysis (PCA; Fig. 1b)^29^. Differences between iBAT and the other tissues were captured by PC1, likely reflecting the effect of developmental time and differentiation status of iBAT, while PC2 captured the origin of the tissues. Comparison of differentially expressed genes (DEG) that exhibited at least a 1.5-fold difference in expression between controls and mutants with a padj < 0.05 indicated that shared changes in gene expression were not frequent in tissues presenting phenotypes in *Hoxa5* mutants (Fig. 1c). The sole gene common to all tissues was *Hoxa5*, which validated the experiment. Two more genes were found as common targets for the tissues of the respiratory system at E15.5, *Gm19248*, a thymosin beta 10 pseudogene, and *Ptprb*, which encodes the protein tyrosine phosphatase receptor type B (Fig. 1d). The transcriptional impact of the *Hoxa5* mutation differed in lung from different ages with only eight genes in common. Finally, *Hoxa5* was the single gene shared by the tissues having somitic origin. Altogether, RNA-seq data support the notion that HOXA5 exerts its functions via the regulation of context-specific effectors. A more detailed analysis of the data will be presented elsewhere.

### *Hox* gene expression in embryonic tissues

It was reported that cross- and auto-regulation participate in the control of intricate and coordinated *Hox* expression, suggesting that *Hox* expression patterns are interdependent^30–35^. To further explore the transcriptomic outputs of *Hoxa5* in different contexts, we thus surveyed the RNA-seq data for changes in global *Hox* gene expression. Such an approach can reveal differences even when individual genes do not reach the threshold cutoff applied above. Interestingly, we observed changes in expression levels of other *Hox* genes across tissues.

**Fig. 2:**
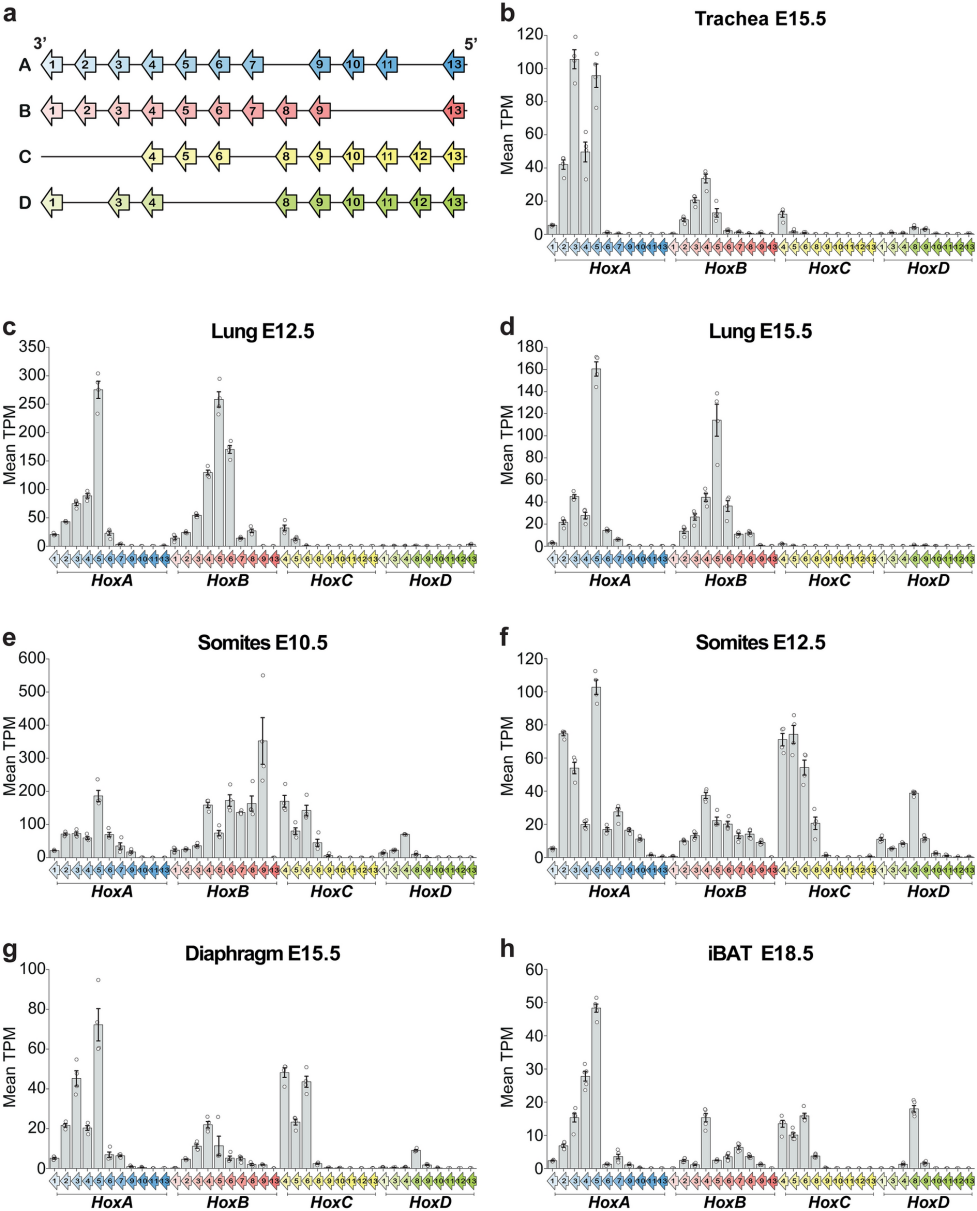
*Hox* gene expression in different tissues during mouse development. (**a**) Schematic representation of the 39 *Hox* genes along the four *Hox* clusters. Arrows represent each *Hox* gene and show the 5ʹ–3ʹ direction of transcription. (**b**–**h**) Mean TPM (transcripts per million) for the 39 *Hox* genes in the indicated tissues and stages in wt embryos. Means were obtained from tissues of four wt embryos for the respiratory system tissues and somites (**b**–**g**) and five wt embryos for iBAT (**h**). Individual data, mean and standard error of the mean (sem) are indicated.

To provide context for these results, we first assessed *Hox* transcript levels in wt tissues (Fig. 2). In trachea and lung, transcripts from the 3’ half of *HoxA* and *HoxB* clusters were the most abundantly expressed (Fig. 2a-d). In trachea, *Hoxa1* to *Hoxa5* expression predominated (Fig. 2b), while in lung, a larger portion of *HoxA* cluster, from *Hoxa1* to *Hoxa7,* was expressed (Fig. 2c, d). Similarly, *Hoxb2* to *Hoxb6* were transcribed in trachea while expression from *Hoxb1* to *Hoxb8* was detected in lung. *HoxC* expression, primarily *Hoxc4* and *Hoxc5,* was observed at much weaker levels in trachea and lung. Likewise, few *HoxD* genes were faintly expressed in the developing trachea (*Hoxd8*-*Hoxd9*) and lung (*Hoxd13*). While there was some variation in relative transcript abundance across timepoints, the same set of *Hox* transcripts was detected in lung at E12.5 and E15.5 (Fig. 2c, d).

A much broader group of *Hox* transcripts was detected in C3-T2 somites (Fig. 2e, f). This region corresponds to the most-anterior domain of *Hoxa5* expression where the homeotic transformations are observed in mutants^15^. In all four *Hox* clusters, the set of genes expressed in somites largely corresponded to previously published expression borders^36,37^. However, several genes whose somitic boundaries lie posterior to T2 were also detected, albeit at low levels. At E10.5 and E12.5, *Hoxa1* to *Hoxa11*, *Hoxb1* to *Hoxb9, Hoxc4* to *Hoxc9* and *Hoxd1* to *Hoxd10* were expressed. Remaining *Abd-B-*family paralog groups were silent. The detection of *Hox9* paralog transcripts may indicate a low level of expression in C3-T2 somites or may reflect expression in the forelimb field. Although the forelimbs were excised, samples likely contained some lateral plate mesoderm-derived progenitors of the forelimb girdle known to express *Hox9* paralog genes^38^. As for the lung, similarities in *Hox* gene expression were observed between somite samples at E10.5 and E12.5, suggesting that *Hox* cluster transcriptional patterns were stably maintained over time.

*Hox* expression in the diaphragm at E15.5 and iBAT at E18.5 followed a pattern related to that seen in somites (Fig. 2g, h). Roughly the same combination of *Hox* genes was expressed in these somite-derived tissues, the diaphragm showing a relatively higher abundance of the more anterior *Hox* genes consistent with its origin from the most anterior part of the sampled somite domain (C3-C5). *Hoxd8*, which is by far the most highly expressed *HoxD* cluster gene in iBAT, is also the highest *HoxD* gene expressed in somites at E12.5. This could reflect an early *Hox* gene activation in somitic BAT progenitors at this stage.

In summary, RNA-seq data revealed a *Hox* expression pattern that varies among tissues but correlates with their embryonic origin. Further, the group of *Hox* genes expressed is largely consistent within a tissue across timepoints. Finally, in all tissues analyzed, *Hoxa5* is among the most highly expressed *Hox* genes.

### *Hoxa5* null mutation perturbs *Hox* gene expression

**Fig. 3:**
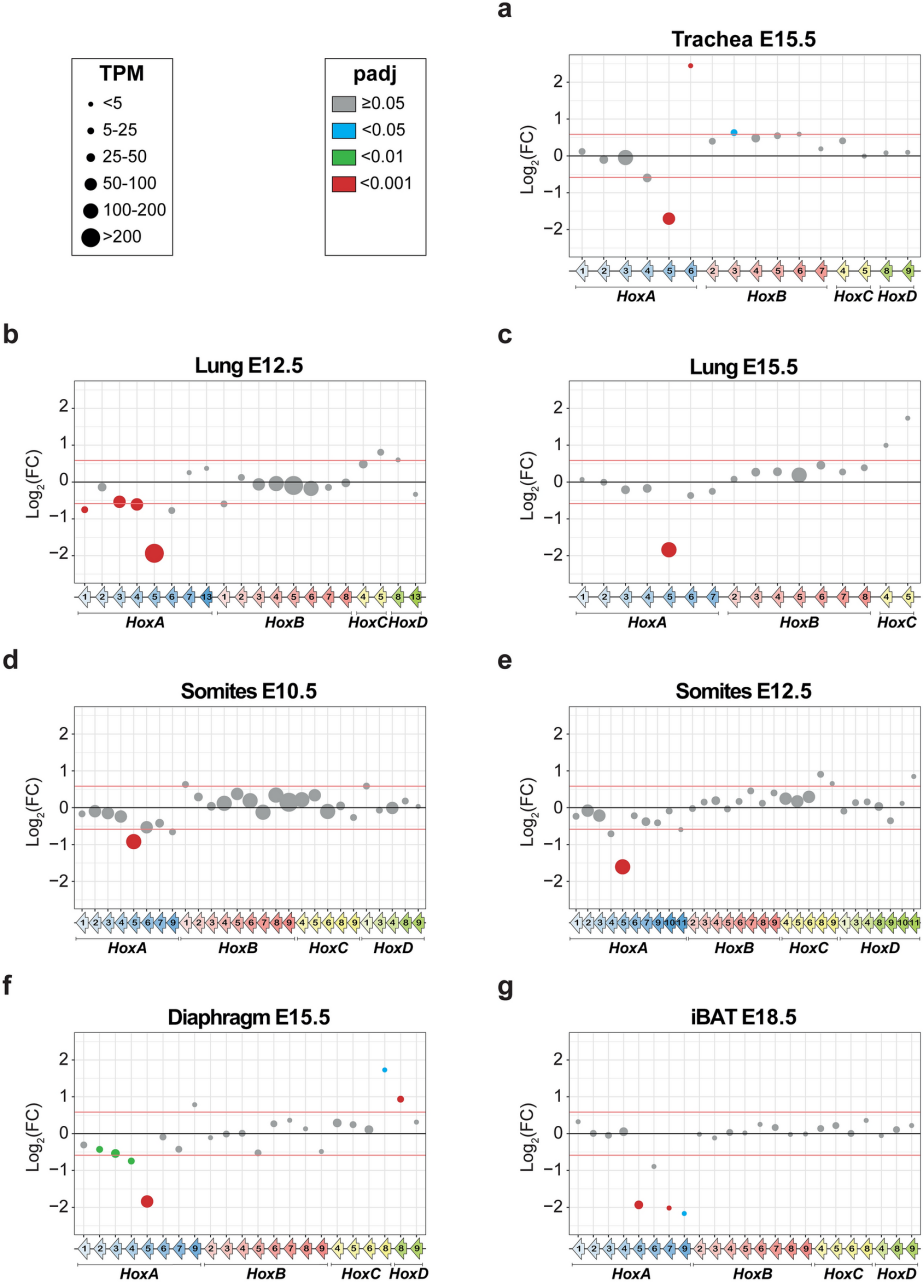
Comparative RNA-seq analysis of *Hox* gene expression between wt and *Hoxa5*^-/-^ mutant conditions. (**a**–**g**) RNA-seq data of *Hox* gene expression in the indicated tissues and embryonic stages. Only *Hox* genes with a mean of TPM ≥ 1 in at least one condition (wt or *Hoxa5*^-/-^) are shown. TPM for each *Hox* gene in wt condition are represented by dot size and padj value by dot color (grey, padj ≥ 0.05; blue, padj < 0.05; green, padj < 0.01; red, padj < 0.001). Differential expression analysis was performed with DESeq2 with an *alpha* parameter of 0.05. A cut-off of 1.5 FC and a padj < 0.05 were used to establish statistically significant differential expression between wt and *Hoxa5*^-/-^ conditions. The Log_2_ value of 1.5 FC is shown by red lines corresponding to -0.58496250 and 0.58496250.

A comparison of *Hox* transcript expression levels in wt versus *Hoxa5*^-/-^ null samples revealed a trend of broad mis-regulation of *Hox* expression, suggesting possible regulation by HOXA5 (Fig. 3). Except for the statistically significant decrease of *Hoxa5* expression in all tissues, the impact of the *Hoxa5* mutation was specific to each tissue and timepoint. In trachea, genes from the *HoxA* and *HoxB* clusters, *Hoxa4*, *Hoxa5*, *Hoxa6*, *Hoxb3* and *Hoxb6*, presented at least a 1.5-fold expression change but the difference was significant only for *Hoxa5*, *Hoxa6* and *Hoxb3* (Fig. 3a). In lung at E12.5, decreased expression of *Hoxa1*, *Hoxa3*, *Hoxa4*, *Hoxa5*, *Hoxa6* and *Hoxb1* was seen in *Hoxa5*^-/-^ mutants while *Hoxc5* and *Hoxd8* levels were augmented. The differences were significant for *Hoxa1*, *Hoxa3*, *Hoxa4* and *Hoxa5*. Three days later, changes in *Hox* lung expression were more limited with only *Hoxa5* showing a significant reduction, while *Hoxc4* and *Hoxc5* expression increased not significantly in *Hoxa5*^-/-^ mutants (Fig. 3b, c). At E10.5 and E12.5, the trends were similar in somites with only *Hoxa5* presenting a significant decrease in transcript levels. Expression of *Hoxa4*, *Hoxa9* and *Hoxa11* was diminished in mutants whereas *Hoxb1*, *Hoxc8*, *Hoxc9*, *Hoxd1* and *Hoxd11* showed at least a 1.5-fold expression increase depending on the embryonic age. However, none of these changes were statistically significant (Fig. 3d, e). In diaphragm, *Hoxa4* and *Hoxa5* were less expressed in *Hoxa5*^-/-^ mutants while *Hoxa9*, *Hoxc8* and *Hoxd8* genes were more expressed. All these differences were significant except for *Hoxa9* (Fig. 3f). Finally, for iBAT, *Hoxa5*, *Hoxa6*, *Hoxa7* and *Hoxa9* genes were less expressed in mutants, and except for *Hoxa6*, these differences were significant (Fig. 3g).

**Fig. 4:**
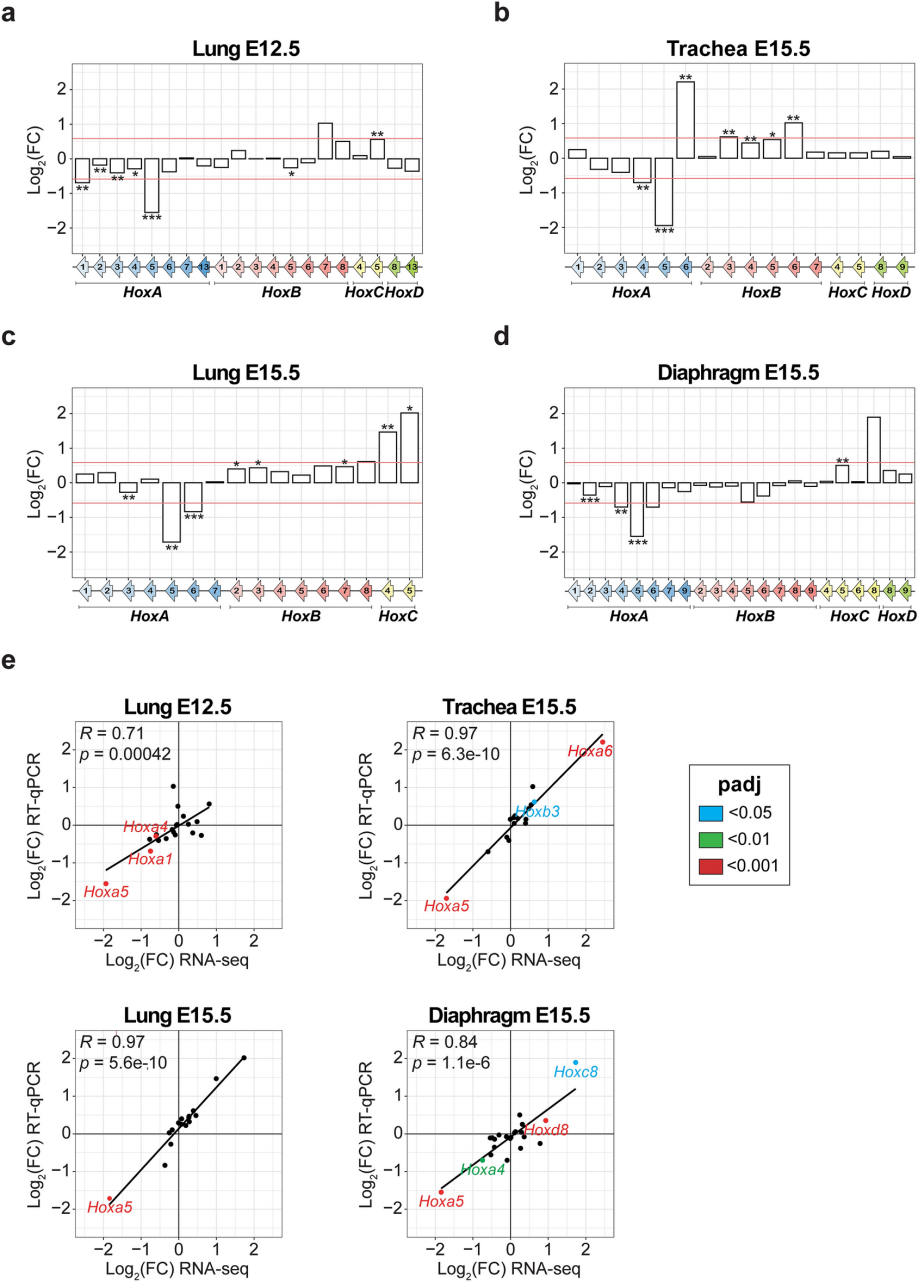
Comparative RT-qPCR analysis of *Hox* gene expression between wt and *Hoxa5*^-/-^ mutant conditions. (**a**–**d**) RT-qPCR analysis of *Hox* gene expression in the indicated tissues and embryonic stages. Five specimens were used for each genotype. Only *Hox* genes with a mean of TPM ≥ 1 in at least one condition (wt or *Hoxa5*^-/-^) and detected in RT-qPCR assays are shown. Student’s *t*-test was performed and a *p* value < 0.05 was considered statistically significant. A cut-off of 1.5 FC and *p* value < 0.05 were used to establish statistically significant differential expression. The Log_2_ value of 1.5 FC is shown by red lines corresponding to -0.58496250 and 0.58496250. *p < 0.05, **p < 0.01, ***p < 0.001. (**e**) Pearson correlation analysis between RNA-seq and RT-qPCR Log_2_(FC) of values of *Hox* gene expression for trachea, lung and diaphragm. The coefficient of correlation (*R*), the *p* value and the linear regression are indicated for each condition. Differentially expressed *Hox* genes identified by RNA-seq with a FC ≥ 1.5 and padj < 0.05 are indicated in color.

To validate the RNA-seq results, quantitative RT-PCR (RT-qPCR) assays were performed for tissues from the respiratory system (trachea E15.5, lung E12.5 and E15.5, and diaphragm E15.5 from wt and *Hoxa5*^-/-^ embryos; Fig. 4a–d). Expression levels obtained by RNA-seq were significantly positively correlated to those determined by RT-qPCR as shown by the Pearson’s correlation coefficient (*R* = 0.71 to 0.97, *p* < 0.0005; Fig. 4e).

Altogether, the data indicated a common and mainly negative effect of the loss of *Hoxa5* function on the expression of *HoxA* genes in all contexts. Depending on the tissue, few genes from the different clusters showed also increased expression. However, the biological impact of these higher transcript levels should be questioned due to their very low expression levels in the different tissues analyzed.

### Impact of *Hoxa5 *mutant alleles on the expression of *HoxA* genes

**Fig. 5:**
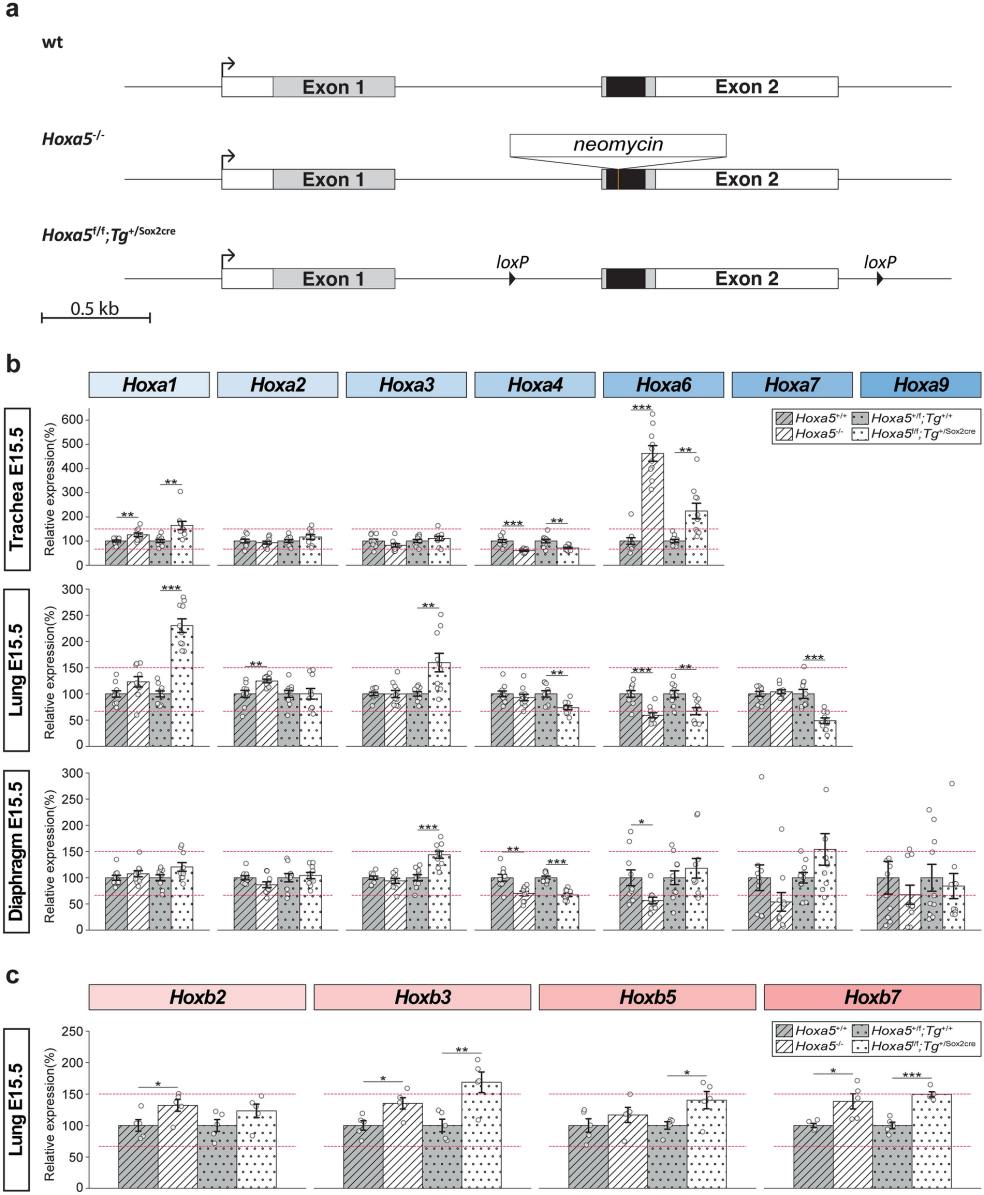
Impact of two different *Hoxa5* null mutations on *HoxA* gene expression. (**a**) Schematic representation of the *Hoxa5* wt, null and conditional alleles. The *Hoxa5* null allele was produced by the insertion of a *neomycin* cassette of 1 kb into the homeobox sequence (black). The *Hoxa5* conditional allele was generated by the insertion of *loxP* sites flanking *Hoxa5* exon 2. Action of the Cre recombinase on the conditional allele deletes exon 2 sequences and generates a null allele. The *Sox2cre* mouse line was used to delete exon 2 in all embryonic tissues. Transcribed and translated sequences are represented by white and grey boxes, respectively. (**b**) Comparative RT-qPCR analyses for *HoxA* genes between the two mutant alleles are shown for trachea, lung and diaphragm of E15.5 mouse embryos. Only *Hox* genes with a mean of TPM ≥ 1 in at least one condition (wt or *Hoxa5*^-/-^) are shown. Ten specimens were used for each genotype. (**c**) Comparative RT-qPCR analyses for *HoxB* genes between the two mutant alleles are shown for lung of E15.5 mouse embryos. Five specimens were used for each genotype. *Hoxa5*^-/-^ specimens are represented by white hatched bars and *Hoxa5*^f/f^;*Tg*^+/Sox2cre^ by white dotted bars. Controls for each mouse line are shown by grey bars. Individual data, mean and standard error of the mean (sem) are indicated. The value of % of 1.5 FC is shown by red lines representing 66.67% and 150%. Student’s *t*-test was performed between each mutant strain and their respective control line, and a *p* value < 0.05 was considered statistically significant. *p < 0.05, **p < 0.01, ***p < 0.001.

The global negative impact of the loss of *Hoxa5* function on flanking *HoxA* genes raised questions about the mechanisms through which the *Hoxa5* mutation acts. One possibility is that the effect is mediated by the HOXA5 protein, either by direct binding to regulatory DNA elements of *Hox* targets, or indirectly by acting on effectors that then control *HoxA* gene expression. Alternatively, the *Hoxa5* null mutant allele could perturb *HoxA* transcription in *cis*, since it contains a 1 kb *neomycin* cassette inserted into the homeobox sequence of the second exon (Fig. 5a)^39^. We have generated a *Hoxa5* conditional mouse line, which once bred with the epiblast-specific *Sox2cre* deleter line, produces a *Hoxa5* null allele as shown by molecular and phenotypic analyses (Fig. 5a)^21,25^. Cre activity on the *Hoxa5* conditional allele deletes a 1.8 kb DNA fragment encompassing the second exon plus few nearby sequences. Either addition or deletion of sequences can interfere with the expression of neighbouring genes^40,41^. We reasoned that effects mediated by the HOXA5 protein should be shared by both mutant alleles. However, while either allele might act in *cis,* their effects could be distinct given that *Hox* genes are tightly packed in *HoxA* cluster. Thus, differences in results between the two mutant alleles could point toward a *cis* effect.

Comparative RT-qPCR analyses between the two mutant lines were performed for trachea, lung and diaphragm from E15.5 wt, *Hoxa5*^-/-^ and *Hoxa5*^f^^/f^; *Tg*^+/Sox2cre^ embryos for *HoxA* genes with a TPM ≥ 1 in at least one condition (wt or *Hoxa5*^-/-^). For trachea, *Hoxa5*^f^^/f^; *Tg*^+/Sox2cre^ specimens entirely recapitulated the changes in *HoxA* expression seen in *Hoxa5*^-/-^ mutants (Fig. 5b). For lung, differences were observed for *Hoxa1* and *Hoxa3*, which were up-regulated in *Hoxa5*^f^^/f^; *Tg*^+/Sox2cre^ specimens when compared to *Hoxa5*^-/-^ mutants, and *Hoxa4* and *Hoxa7*, which were down-regulated in *Hoxa5*^f/f^; *Tg*^+/Sox2cre^ samples. However, *Hoxa4* decreased expression was less than 1.5-fold change. In diaphragm, *Hoxa5*^f/f^; *Tg*^+/Sox2cre^ specimens largely reproduced the data obtained with *Hoxa5*^-/-^ samples, except for *Hoxa3*, which showed an increased expression that was less than 1.5-fold change in *Hoxa5*^f/f^; *Tg*^+/Sox2cre^ embryos.

Overall, the two *Hoxa5* mutations caused largely similar effects on *HoxA* gene expression in the three tissues, with few exceptions in lung. The lack of consistent differences in the outcome of the two mutations across tissues, as it would be expected from a *cis*-acting effect, does not support transcriptional interference on the nearby genes even though the possibility cannot be excluded. Rather, the two *Hoxa5* mutations presented the same tendency, which favored the notion that the impact of the loss of *Hoxa5* function on the expression of flanking *HoxA* genes is due to the lack of the HOXA5 protein. Comparative RT-qPCR assays for *Hoxb2*, *Hoxb3*, *Hoxb5* and *Hoxb7* genes showed similar increased expression in *Hoxa5*^-/-^ and *Hoxa5*^f/f^; *Tg*^+/Sox2cre^ mutants when compared to controls further supporting the *trans*-acting action of HOXA5 on the expression of *Hox* genes (Fig. 5c).

### Effect of *Hoxa5 *mutant alleles on the *HoxA* chromatin landscape

**Fig. 6:**
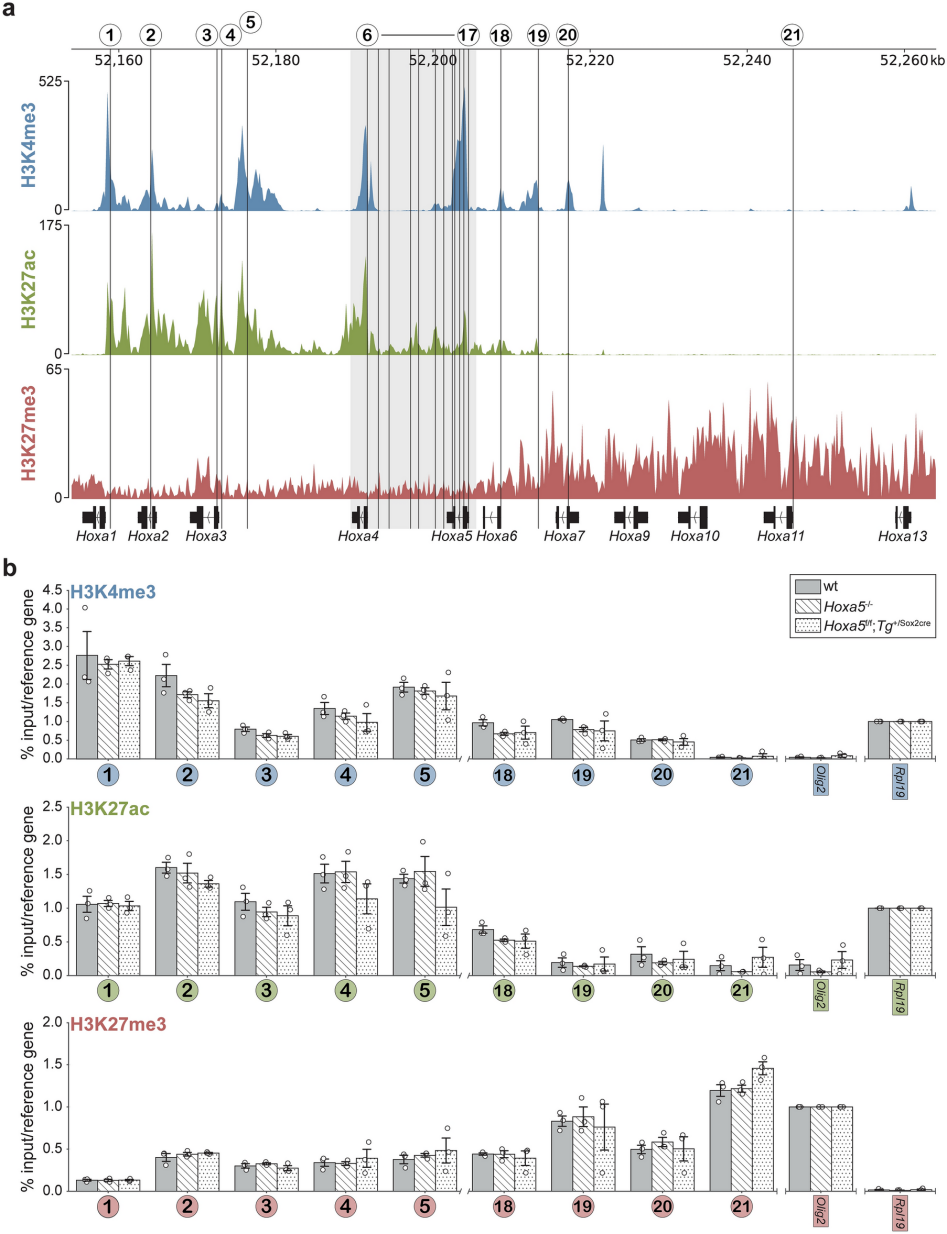
Analysis of epigenetic marks along the *HoxA* cluster from E15.5 wt, *Hoxa5*^-/-^ and *Hoxa5*^f/f^;*Tg*^+/Sox2cre^ mouse lung tissue. (**a**) ChIP-seq profiles for H3K4me3 and H3K27ac positive histone marks, in blue and green, respectively, and H3K27me3 repressive mark, in red, along the *HoxA* cluster in lung from E15.5 wt mouse embryo were used as a reference. They were obtained from the ENCODE Project^46^. The mm38 mouse genome reference (NCBI) was used for manual curation and accurate representation of *Hox* genes. Numbers and vertical lines refer to regions (1 to 21) along the *HoxA* cluster analyzed by ChIP-qPCR. Enlargement of the *Hoxa5* locus (regions 6 to 17) is shown separately in Fig. 7. (**b**) Individual data, mean and standard error of the mean (sem) from three independent chromatin isolation and ChIP assays for lung of wt (grey bars), *Hoxa5*^-/-^ (white hatched bars) and *Hoxa5*^f/f^;*Tg*^+/Sox2cre^ (white dotted bars) E15.5 mouse embryos are shown. ChIP-qPCR assays for each region are represented for H3K4me3, H3K27ac and H3K27me3 histone marks. The numbers below each group correspond to the regions shown in (a). The % input for each mark was normalized by the % input of a reference DNA region. *Rpl19* DNA region was used to normalize the data of H3K4me3 and H3K27ac while *Olig2* was used to normalize the data of H3K27me3. No statistically significant differences (padj < 0.05) were observed by one-way ANOVA test.

**Fig. 7:**
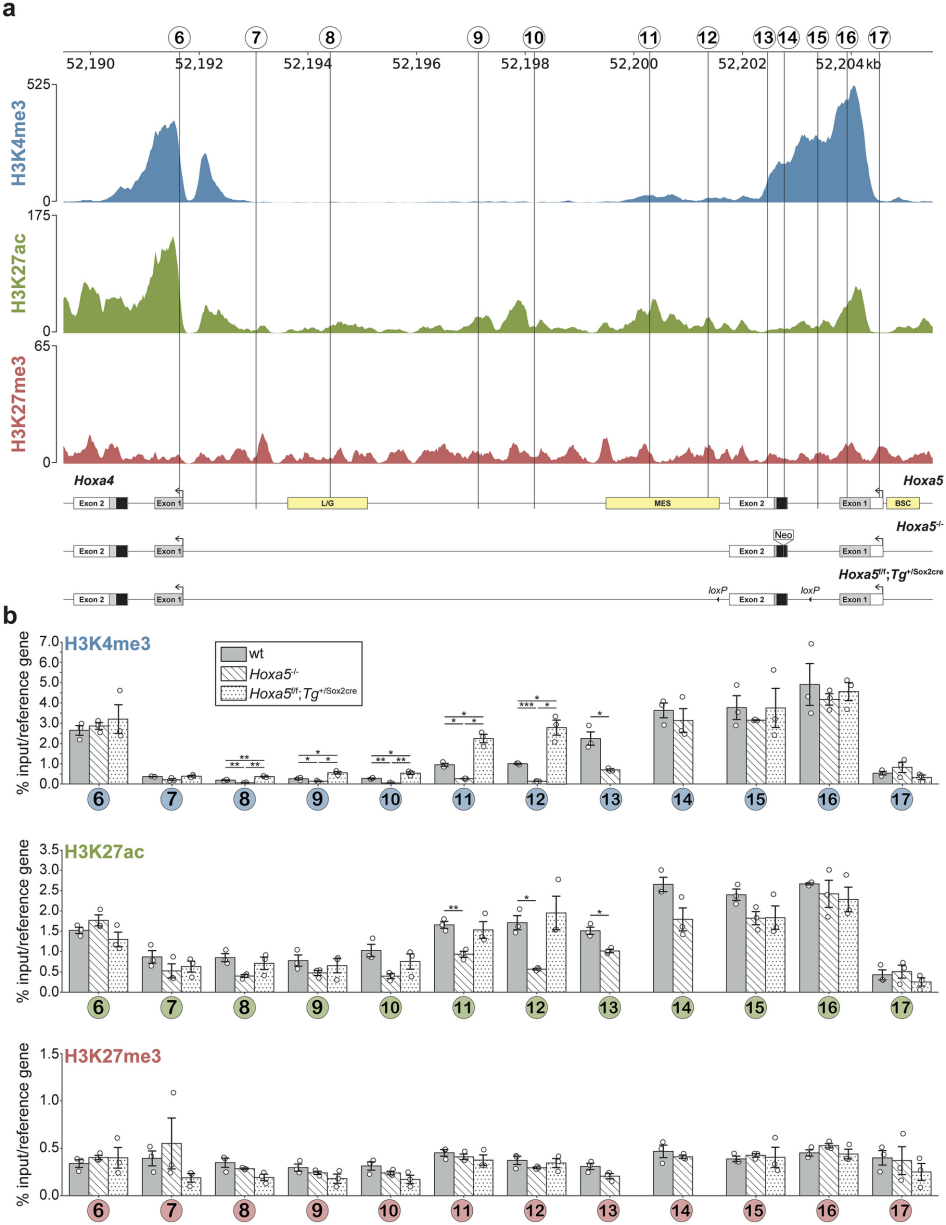
Analysis of epigenetic marks along the *Hoxa5* locus from E15.5 wt, *Hoxa5*^-/-^ and *Hoxa5*^f/f^; *Tg*^+/Sox2cre^ mouse lung tissue. (**a**) ChIP-seq profiles for H3K4me3 and H3K27ac positive histone marks and H3K27me3 repressive mark, at the *Hoxa5* locus in E15.5 wt mouse lung tissue, obtained from ENCODE^46^. Numbers and vertical lines refer to regions (6 to 17) analyzed by ChIP-qPCR. The three *Hoxa5* alleles are represented beneath the profiles. Black, grey and open boxes represent homeobox, translated and transcribed sequences, respectively. Transcription start sites are shown by black arrows. For the wt allele, yellow boxes define DNA regulatory sequences driving *Hoxa5* tissue-specific expression: BSC, brachial spinal cord enhancer; MES, mesodermal enhancer; L/G, lung and gut enhancer (Jeannotte et al., 2016). For the *Hoxa5* null allele, the 1 kb MC1neoA^+^ cassette inserted into the homeobox is represented. (**b**) Individual data, mean and standard error of the mean (sem) from three independent chromatin isolation and ChIP assays for lungs of wt (grey bars), *Hoxa5*^-/-^ (white hatched bars) and *Hoxa5*^f/f^;*Tg*^+/Sox2cre^ (white dotted bars) E15.5 mouse embryos are shown. ChIP-qPCR assays for each region are represented for H3K4me3, H3K27ac and H3K27me3 histone marks. The numbers below each group correspond to the regions shown in A. The % input for each mark was normalized by the % input of a reference gene. *Rpl19* was used to normalize the data of H3K4me3 and H3K27ac while *Olig2* was used to normalize the data of H3K27me3. Student’s *t*-test with correction for False Discovery Rate (FDR) was applied for regions statistically significant in one-way ANOVA test. A padj < 0.05 was considered statistically significant. *padj < 0.05, **padj < 0.01, ***padj < 0.001.

The tight organization of *Hox* genes into clusters suggested that the effects of the loss of *Hoxa5* function on *HoxA* gene expression could result from chromatin organization changes that affect all or part of the *HoxA* cluster. To test this hypothesis, we analyzed the chromatin landscape of the *HoxA* complex in lung from E15.5 wt, *Hoxa5*^-/-^ and *Hoxa5*^f/f^; *Tg*^+/Sox2cre^ embryos. Chromatin profiling was performed for three histone post-translational modifications (PTM) associated either with transcriptional activation or repression. H3K27ac is found on promoters and enhancers of transcribed genes, H3K4me3 is present on promoters of active genes and bivalent domains, while H3K27me3 marks non-expressed genes and bivalent domains^42–45^. Genome-wide ChIP-seq data available from ENCODE displayed patterns of histone modifications in lung from E15.5 wt mouse embryos^46^. Comparison of the profiles for the three histone PTMs with our transcriptomic data showed that the chromatin state along the *HoxA* cluster predicts the abundance of *HoxA* transcripts in lung (Figs. 2d, 6a). Positive histone marks were found in the 3ʹ half of the cluster that contains *HoxA* genes expressed in the developing lung. The H3K27me3 repressive mark, although distributed across the entire *HoxA* cluster, was enriched in the 5ʹ region of the cluster where *Hox* genes show no lung expression. The transition occurred in the *Hoxa5*-*Hoxa7* region, and the pattern of peaks for the positive marks was complementary to that of H3K27me3. Finally, the H3K27ac active mark was present in *Hoxa5* regulatory enhancers, previously identified by transgenesis and located in the *Hoxa4*-*Hoxa5* intergenic region, that drive expression in embryonic lung and gut, and in mesoderm derivatives. (Fig. 7a)^47,48^.

To determine if the addition of the *neomycin* cassette in *Hoxa5*^-/-^ mutant lungs or the deletion of exon 2 sequences in *Hoxa5*^f/f^; *Tg*^+/Sox2cre^ specimens perturb *HoxA* chromatin organization, we analyzed by ChIP-qPCR the effect of the two mutations on the profile of the three histone PTMs for 21 regions covering the *HoxA* cluster (Figs. 6a, 7a). These regions largely matched the peaks of H3K4me3 and H3K27ac positive marks corresponding to the transcription start site of *HoxA* genes and to *Hoxa5* enhancer sequences. One exception was region 21 only enriched for the H3K27me3 repressive mark. Interestingly, ChIP-qPCR data obtained for the three marks correlated well with the ENCODE ChIP-seq study. Both datasets indicated that the positive marks were higher for *HoxA* genes from the 3ʹ half of the cluster (regions 1 to 6; Figs. 6b, 7b). Conversely, genes from the 5ʹ part of the cluster showed a stronger ChIP-qPCR signal for H3K27me3 in agreement with the transcriptional inactivity of these genes in the developing lung (regions 19–21; Fig. 6b). For regions 1 to 6, which covers *Hoxa1* to *Hoxa4* genes, no difference was seen between wt and the two *Hoxa5* mutant specimens. A similar result was obtained for regions 18 to 21 that overlap *Hoxa6* to *Hoxa11* genes (Fig. 6b). Thus, neither *Hoxa5* mutations perturbed the chromatin landscape of the flanking *HoxA* genes based on the histone PTMs tested.

The observed effects of *Hoxa5* mutation on *HoxA* expression could result from limited chromatin perturbations in shared regulatory elements close to the targeted mutation. Indeed, co-regulation of clustered *Hox* genes is known to be mediated by shared *cis-*regulatory elements, although these have not been fully defined for the 3ʹ half of *HoxA* cluster. To characterize local effects on chromatin landscape flanking *Hoxa5*, the *Hoxa5* gene was dissected into 11 regions (regions 7 to 17; Fig. 7a). The site of insertion of the *neomycin* cassette is located downstream region 14, while the deletion of exon 2 sequences includes regions 13 and 14. ChIP-qPCR data for the three marks showed no significant difference for regions 14 to 17 indicating that neither DNA insertion nor deletion impacted *Hoxa5* gene and promoter sequences located upstream both mutations (Fig. 7b). Similarly, ChIP-qPCR data for the H3K27me3 repressive mark revealed no variation between wt and the two *Hoxa5* mutations in regions 7 to 17. In contrast, significant differences were seen for the H3K4me3 and H3K27ac positive histone PTMs in regions located downstream the *Hoxa5* mutations, and each mutation elicited a specific response. For the H3K27ac mark, decreased ChIP-qPCR signal was detected in regions 11, 12 and 13 in *Hoxa5*^-/-^ mutants but no change was seen in *Hoxa5*^f/f^; *Tg*^+/Sox2cre^ specimens. For the H3K4me3 mark, the effect of the *Hoxa5* null mutation was much broader than for H3K27ac and diminished ChIP-qPCR signal extended into the *Hoxa4*-*Hoxa5* intergenic sequence covering regions 8 to 13. Conversely, the *Hoxa5*^f/f^; *Tg*^+/Sox2cre^ conditional mutation caused an increased ChIP-qPCR signal for H3K4me3 for regions 8 to 12. Thus, either the insertion of sequences into *Hoxa5* exon 2 or the deletion of *Hoxa5* exon 2 sequences have limited influence on the chromatin landscape of *HoxA* cluster, the effects being restricted to marks associated with active chromatin on *Hoxa5* downstream sequences nearby the gene.

## Discussion

To tackle the mechanisms of action of HOXA5, we addressed its transcriptional impact in a genome-wide scale by applying RNA-seq to seven different biological contexts in wt and *Hoxa5*^-/-^ embryos. Our initial hypothesis was that HOXA5 exerts part of its functions through activation of common transcriptional programs but also via the regulation of distinct, context-specific effectors. According to the results obtained, this hypothesis was not supported. Indeed, *Hoxa5* was the only DEG shared by all conditions analyzed. Even for a same organ at different timepoints, the overlap in DEG was modest, which may reflect unique roles for HOXA5 throughout organogenesis. For tissues at late developmental stages, like iBAT at E18.5, it may also suggest indirect consequences of earlier effects of the loss of *Hoxa5* function. Altogether, the results indicate that HOXA5 transcriptional output depends on its site and time of expression.

While this result was surprising, *Hox* genes are known to exert subtle effects on tissue development, and to regulate subsets of cells within tissues. Subtly affected transcripts would not be expected to reach the thresholds for inclusion in the DEG analysis. Comparison of RNA-seq data with earlier RT-qPCR experiments validates this notion. For instance, no significant change in *Hoxb5* lung expression was detected in *Hoxa5*^-/-^ specimens as it was previously reported (Fig. 3)^20^. Similarly, changes in expression of several genes in trachea, lung, and diaphragm, as described in^21^, were also found in the RNA-seq analysis even though the variations in expression levels did not reach the 1.5-fold change limit. Bulk RNA-seq experiments on whole tissue do not capture cell-specific data. In the different biological contexts tested here, *Hoxa5* expression is often heterogenous. HOXA5 protein is specifically detected in the mesenchyme along the respiratory tract, while in diaphragm HOXA5 is found in pleuroperitoneal folds and their derivatives but not in the muscle cell lineage originating from somites^21^. In somites, HOXA5 expression is mainly restricted to sclerotome^49^. HOXA5 is largely expressed in BAT connective tissue fibroblasts at the stage surveyed^23^. While bulk RNA-seq approach has the advantage to reveal both HOXA5 direct targets and indirect downstream effectors in neighbouring tissues, to specifically address tissue heterogeneity and diversity, single cell RNA-seq assay will need to be applied.

One explanation for the context-specific dependence of HOXA5 action may come from the cooperative association of HOX proteins with co-factors, like the three amino acid loop extension (TALE) homeodomain transcription factors, PBX, MEIS and PREP^11^. TALE proteins are important in DNA binding, target specificity and recruitment of other cofactors, leading to the formation of multi-protein complexes that define the functional outcome of HOX binding^8,13^. Few HOXA5 interactors have been identified so far and none for the tissues tested in this study^50–52^. As each TALE protein possesses a cell-specific expression pattern, this may contribute to the diversity of transcriptional programs controlled by HOXA5. Indeed, our RNA-seq data showed that the combination of TALE factors expressed in the different biological contexts is distinct for each condition. For example, only *Pbx2* is expressed in iBAT, *Pbx4* is weakly expressed in all tissues analyzed, and levels of expression for most TALE proteins are lower in lung at E15.5 than at E12.5. Further studies are needed to identify which HOXA5 partners participate in its context-specific activity and how they act together.

*Hox* transcriptional initiation depends on long-range global enhancers flanking clusters, local enhancers within clusters, as well as Topologically Associated Domains (TAD) structures that permit fine control over chromatin contacts and patterns of histone modifications^4,53,54^. It is unclear whether and how HOX proteins function during the establishment of *Hox* cluster transcription. However, later functions for HOX proteins in cross- and auto-regulation have been demonstrated^8,30–35^. The trend for a widespread mis-regulation of *Hox* expression in *Hoxa5* mutants supports a role for HOXA5 in the control of *Hox* gene expression independent of tissue and time contexts. The loss of *Hoxa5* function mainly causes reduced expression levels of a subset of *HoxA* transcripts in most conditions tested. No clear trend for the *HoxB* cluster was observed while affected *HoxC* and *HoxD* genes showed increased expression in *Hoxa5* mutant specimens. Except for *Hoxd8* in lung, these genes were already expressed in wt samples albeit at low levels. Thus, while the expression levels of several *Hox* genes was altered in a direction that largely correlated with cluster, the combination of *Hox* genes expressed in each condition remained unchanged in *Hoxa5* mutants. This suggests that the loss of *Hoxa5* function does not affect the establishment or maintenance of global *Hox* chromatin structure such as the location of TAD boundaries or enhancer contacts, which are proposed to determine the set of *Hox* genes that can be expressed in a tissue^53^. To our knowledge, this is the first demonstration of widespread changes in *Hox* expression levels following mutation of a single protein coding *Hox* gene.

It is also largely undefined whether targeted genome modifications used to disrupt single *Hox* genes affect *Hox* cluster expression in *cis*. The comparative expression analysis of the impact of two *Hoxa5* mutant alleles revealed a similar trend in *HoxA* gene expression variation supporting that the two distinct *Hoxa5* mutations did not interfere in *cis* on the expression of *HoxA* genes. One exception is the increase in *Hoxa3* expression in lung and diaphragm, but not trachea, of *Hoxa5*^f/f^; *Tg*^+/Sox2cre^ embryos indicating that a possible tissue-specific *cis*-effect may occur (Fig. 5b). However, the shared anomalies resulting from the two *Hoxa5* null alleles argue against the possibility that this potential *cis* effect contributes substantially to the *Hoxa5* mutant phenotypes.

Pervasive *Hox* transcriptional changes were reported following deletions of *Hox-*embedded microRNA genes, an observation ascribed at least in part to direct regulatory interactions between *Hox* miRNAs and their coding *Hox* gene targets^55^. Therefore, it is possible that additional mechanisms exist and allow compensatory changes to global *Hox* transcription. For instance, the broad mis-regulation of *Hox* gene expression seen in *Hoxa5* mutants could be a secondary response to the mis-expression of few HOXA5 primary *Hox* target genes.

In vertebrates, functional redundancy among *Hox* paralogs has been demonstrated, and in some situations, cross-transcriptional regulation within a paralog group has been shown to be an important mechanism for controlling *Hox* dosage^2,31^. In the case of *Hoxa5*, RNA-seq data did not show evidence for compensatory expression changes in *Hoxb5* and *Hoxc5* paralogs in the different conditions analyzed (Fig. 3). Moreover, the impact of the loss of *Hoxa5* function involves effects that extend beyond a single paralog group. Regardless of the underlying mechanism(s), our results raise the point that alterations in global *Hox* cluster output should be considered when interpreting the phenotypes of *Hox* mutations.

Several studies have characterized the *HoxA* chromatin landscape, and the histone modifications associated with transcriptional initiation in embryonic stem cells and maintenance in their derivatives^54,56,57^. The *HoxA* cluster was shown to overlap the boundary between two TADs. The switch between the H3K27ac and H3K4me3 positive histone marks and the H3K27me3 repressive mark in the *Hoxa5*-*Hoxa7* region seen for the embryonic lung at E15.5 was also reported in motor neurons where it coincides with highly conserved binding sites for the CCCTC-binding factor CTCF, known to demarcate borders between TADs. Based on these findings, it is tempting to speculate that *Hoxa5* mutations mainly impact expression of *HoxA* genes located in the 3’ half of the cluster due to their proximity within one of the TADs. More studies on the chromatin architecture of *Hox* clusters in developing tissues, including lung, are needed to support this hypothesis.

ChIP-qPCR assays showed that the effects on chromatin of the two *Hoxa5* mutations are confined to *Hoxa5* flanking downstream sequences. For the *Hoxa5*^*-/-*^ null mutation, decreased ChIP-qPCR signal was observed for the H3K4me3 and H3K27ac positive marks, while the *Hoxa5*^f/f^; *Tg*^+/Sox2cre^ conditional mutation caused an increased ChIP-qPCR signal for the H3K4me3 mark (Fig. 7b). In wt specimens, strong peaks of both marks were seen in *Hoxa5* promoter region, and the height of these peaks diminishes along *Hoxa5* exons to conclude with a weak signal for H3K27ac and no signal for H3K4me3 once reaching the *Hoxa4*-*Hoxa5* intergenic region. One likely explanation for the contrasting responses between the two mutations might be that addition of the *neomycin* cassette in the *Hoxa5* null allele moves the sequences downstream the insertion site away from the promoter. This is further supported by the lack of variation between wt and *Hoxa5*^-/-^ specimens in region 14 located immediately upstream the site of insertion of the *neomycin* cassette. Conversely, deletion of exon 2 sequences following the recombinase action on the *Hoxa5* conditional allele brings the sequences downstream of the mutation closer to the promoter. These changes are more noticeable with H3K4me3, a predominant and well-defined mark of promoter sequences, whereas H3K27ac is a broader mark covering promoter and enhancer regions. Altogether, these data suggest that (i) *Hoxa5* targeted mutations do not significantly perturb the establishment and maintenance of the global chromatin structure of *HoxA* cluster, and (ii) changes in *HoxA* gene expression seen in *Hoxa5* mutants are likely mediated by the HOXA5 protein itself. However, to resolve the role of HOXA5 in *Hox* gene regulation, identification of *bona fide* HOXA5 transcriptional targets in different biological contexts on a genome-wide scale is indispensable.

## Methods

### Mouse lines, genotyping, and tissue collection

The *Hoxa5* null (*Hoxa5*^tm1Rob^) and *Hoxa5*^f/f^ (*Hoxa5*^tm1Ljea^) mouse lines were generated by the senior author and her team, while the *Sox2cre* (Edil3^Tg(Sox2-cre)1Amc^) mouse line was directly obtained from Dr. Andrew McMahon^15,25,58^. Mouse lines were maintained in the 129/Sv background. Age of embryos was estimated by considering the morning of the day of the vaginal plug as E0.5. Pregnant females were sacrificed by using isoflurane inhalation and cervical dislocation. Experimental specimens were genotyped by PCR^25,49^. Trachea and diaphragm were collected at E15.5, lungs at E12.5 and E15.5, somites at E10.5 and E12.5, and iBAT at E18.5. For somite samples, the trunk segments located between the C3 and T2 vertebrae were removed and dissected away from the neural tube, forelimbs, and thoracic organs. For RNA extraction, embryonic tissues were dissected into ice-cold PBS, and either homogenized immediately in Trizol (Invitrogen), following manufacturer’s instructions or snap-frozen in liquid nitrogen until further processing. Experiments were performed according to the guidelines of Canadian Council on Animal Care and agreed by the Université Laval Animal Care Committee or approved by the Columbia University IACUC. The study is reported in accordance with ARRIVE guidelines (https://arriveguidelines.org).

### Bulk RNA-seq analysis

For trachea (E15.5), lungs (E12.5 and E15.5), diaphragm (E15.5) and somites (E12.5), four biological replicates were used for both wt and *Hoxa5*^-/-^ genotypes. For somites (E10.5), four wt and three *Hoxa5*^-/-^ mutants were used while for iBAT (E18.5), five samples were used for each genotype. mRNA isolation from total RNA by polyA selection, library preparation and sequencing on Illumina HiSeq were performed at the Columbia University Sulzberger Genome Center (New York, USA) for trachea, lung and diaphragm specimens (100 bp reads, single-end), and at Genewiz (South Plainfield, USA) for somite and iBAT samples (150 bp reads, paired-end).

The quality of the sequencing reads was evaluated using FastQC software (http://www.bioinformatics.babraham.ac.uk/projects/fastqc). Reads were trimmed with Trim Galore (v0.6.5; https://github.com/FelixKrueger/TrimGalore), then mapped with STAR (v2.7.9a)^59^ to the mouse reference genome from ENSEMBL GRCm39 (mm39, release 109)^60^, which was manually curated for *Hox* genes. Between 80 and 94% of the reads mapped successfully to the mouse genome (Supplementary Table S1 online). The mapped sequencing data were then processed with the software featureCounts to obtain counts for sequencing reads mapped to genes (v2.0.3)^61^. Among the aligned reads, 70% mapped to unique genomic regions and they were further considered for analysis (Supplementary Table S1 online). Gene expression levels were represented as transcripts per million (TPM). Genes with a mean of TPM < 1 in both wt and mutant conditions were removed before performing differential expression analysis using DESeq2 (Bioconductor) with an *alpha* parameter of 0.05 (v1.42.0)^62^. Genes were considered to be differentially expressed when they exhibited at least a 1.5-fold difference (fold change; FC) between controls and mutants with a false discovery rate (FDR) adjusted pvalue (padj) < 0.05. The value of a fold change ≥ 1.5 was chosen to take into account the small number of replicates, the established biological variation between specimens and the fold change in *Hoxa5* expression in the different contexts (Supplementary Fig. 1).

### Quantitative RT-PCR (RT-qPCR) assays

RT-qPCR experiments were performed as previously described^19^. Five or ten specimens for trachea, lung and diaphragm were used for each genotype tested. RNA preparations were submitted to a DNase I treatment step since for some *Hox* genes, primers were in the same exon. Primer sequences are listed in Supplementary Table S2 online.

### Chromatin immunoprecipitation (ChIP) and ChIP-qPCR assays

Lung from E15.5 embryos were isolated and cross-linked in 1% formaldehyde prepared in phosphate-buffered saline (PBS) for 10 min at room temperature. Cross-linking was stopped by adding glycine to a final concentration of 125 mM with a 5 min incubation at room temperature. Samples were then washed once into PBS, snap-frozen in liquid nitrogen and kept at −80 °C. Lungs were disrupted with a Dounce homogenizer in Nucleus Isolation Buffer (20 mM HEPES, 250 mM sucrose, 3 mM MgCl_2_, 0.25% Nonidet P-40, 3 mM ß-mercaptoethanol and protease inhibitors), equilibrated on ice for 20 min, and filtered on cell strainer (70 μm) to eliminate debris. Nuclei were lysed in 50 mM Tris–HCl pH8.0, 10 mM EDTA, 1% SDS and protease inhibitors for 10 min on ice. Chromatin was then fragmented by sonication in a Covaris M220 Focused Ultrasonicator to obtain an average DNA size of 200–500 bp. Fragmented chromatin (20 μg) was incubated overnight at 4 °C with 5 μl of antibody: H3K4me3 (Cell Signaling, C42D8), H3K27ac (Cell Signaling, D5E4), or H3K27me3 (Active Motif, 39055) or with 4 μl of IgG (Millipore Sigma, PP64) as control. Immunoprecipitation products were isolated with Dynabeads protein G (ThermoFisher), washed in successive saline buffers, and reverse cross-linked in 200 mM NaCl overnight at 65 °C. Samples were then digested with Proteinase K (0.12 mg/ml) and RNaseA (0.04 mg/ml) in 40 mM Tris–HCl pH8.0, 10 mM EDTA for 2 h at 50 °C.

Purified DNA was analyzed by ChIP-qPCR with primers corresponding to 21 regions covering the *HoxA* complex. *Rpl19* and *Olig2* genes were respectively used as control DNA regions to normalize the H3K27ac and H3K4me3 positive and the H3K27me3 repressive histone marks. According to ENCODE ChIP-seq data, in lung from E15.5 mouse embryo, *Rpl19* and *Olig2* gene regions are respectively enriched in positive and repressive histone marks^46^. Values were reported as the percentage relative to input. ChIP results were confirmed by three independent chromatin isolation and ChIP assays. Each biological replica represents the chromatin from a pool of 7 to 10 lungs. ChIP-qPCR was performed in triplicate for each sample. Primer sequences are listed in Supplementary Table S3 online.

As a reference, we used ChIP-seq data available from ENCODE for lung from E15.5 wt mouse embryos for the three following histone post-translational modifications: H3K27ac, GEO:GSE83004; H3K4me3,GEO:GSE82583; H3K27me3, GEO:GSE82981^46^.

### Statistical analyses

Student’s *t*-test was performed for RT-qPCR assays and a significance level below 5% (*p* value < 0.05) was considered statistically significant. As mentioned above, RNA-seq data were analyzed with DESeq2 and a padj < 0.05 was considered statistically significant. One-way Anova was performed for comparatives studies of ChIP-qPCR data. Student’s *t*-test with correction for False Discovery Rate (FDR - Benjamini, Krieger and Yekutieli method) was applied for regions statistically significant in one-way ANOVA test^63^. A padj < 0.05 was considered statistically significant.

## Supplementary Information

Supplementary Information.

## Data Availability

ChIP-seq data for H3K4me3, H3K27ac and H3K27me3 from ENCODE are available through NCBI GEO with the following accession numbers: H3K27ac, GEO:GSE83004; H3K4me3, GEO:GSE82583; H3K27me3, GEO:GSE82981^46^. RNA-seq data and genome with curated *Hox* genes are available for general access through NCBI GEO (accession number GSE269950). RNA-seq data are now available for general access through NCBI GEO.
