# Supplementary material for: Loss of Hoxa5 function affects Hox gene expression in different biological contexts
Source: Sci Rep. 2024 Dec 28;14:30903. doi: 10.1038/s41598-024-81867-0 (PMC11680909; doi:10.1038/s41598-024-81867-0)
Supplement: Supplementary file 1 — Supplementary Information. [file 41598_2024_81867_MOESM1_ESM.pdf]

Loss of *Hoxa5* function affects *Hox* gene expression in different biological contexts

Béatrice Frenette, Josselin Guéno, Nicolas Houde, Kim Landry-Truchon, Anthony Giguère, Theyjasvi Ashok, Abigail Ryckman, Brian R. Morton, Jennifer H. Mansfield and Lucie Jeannotte

SUPPLEMENTAL DATA

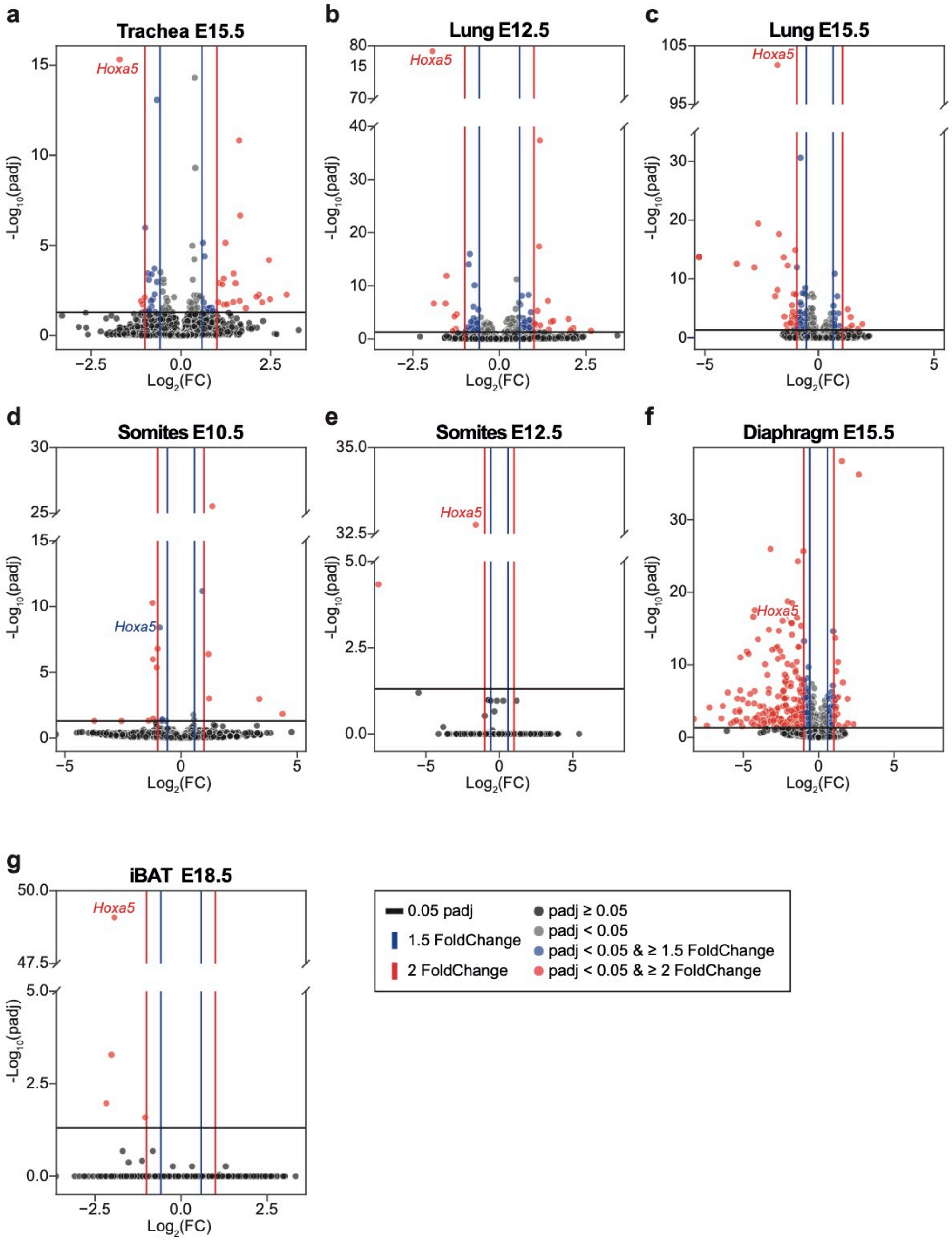

Supplementary Figure 1. Differential gene expression in *Hoxa5*$^{-/-}$ mutant tissues according to Fold Change and padj. Volcano plot representation of differential gene expression analysis between wt and *Hoxa5*$^{-/-}$ for (a) trachea at E15.5, (b) lung at E12.5, (c) lung at E15.5, (d) somites at E10.5, (e) somites at E12.5, (f) diaphragm at E15.5 and (g) iBAT at E18.5. The x-axis shows Log2Fold Change (FC) expression while the y-axis represents the padj in -Log10. Blue and red vertical lines represent a FC threshold of 1.5 and 2, respectively. The horizontal black line corresponds to padj of 0.05. Genes with no statistical change (padj ≥ 0.05) correspond to black dots. Genes with statistical change (padj < 0.05) and with FC < 1.5, ≥ 1.5 or ≥ 2 are represented by gray, blue and red dots, respectively. The *Hoxa5* gene is indicated in each context.

Supplementary Table 1. RNA-seq statistics

Tissue	Genotype	Sample	Reads	Number of reads generated	Number of reads after filtering	Number of uniquely mapped reads	% of reads mapped to genome	% successfully assigned alignments
Lung E12.5	wt	JJ017_S1	Single-end	44912901	44903775	40056350	89.2	66.7
Lung E12.5	wt	JJ018_S2	Single-end	48721446	48707812	43270824	88.84	65.4
Lung E12.5	wt	JJ019_S3	Single-end	37868327	37855884	34023830	89.88	67.4
Lung E12.5	wt	JJ020_S4	Single-end	35407355	35395616	31876236	90.06	68.1
Lung E12.5	Hoxa5-/-	JJ021_S5	Single-end	34185404	34178228	30668022	89.73	64.2
Lung E12.5	Hoxa5-/-	JJ022_S6	Single-end	36088288	36077099	32513452	90.12	67.7
Lung E12.5	Hoxa5-/-	JJ023_S7	Single-end	95201982	95184349	85645271	89.98	67.8
Lung E12.5	Hoxa5-/-	JJ024_S8	Single-end	26878660	26873414	24137646	89.82	67.4
Trachea E15.5	wt	JJ025_S9	Single-end	32311373	32301905	29527039	91.41	70
Trachea E15.5	wt	JJ026_S10	Single-end	41523338	41515444	37826247	91.11	68.2
Trachea E15.5	wt	JJ027_S11	Single-end	52706850	52696828	47986842	91.06	67.9
Trachea E15.5	wt	JJ028_S12	Single-end	30628117	30617213	27878030	91.05	67.8
Trachea E15.5	Hoxa5-/-	JJ029_S13	Single-end	32334476	32323551	29468550	91.17	68.2
Trachea E15.5	Hoxa5-/-	JJ030_S14	Single-end	40427737	40420771	36723262	90.85	67.9
Trachea E15.5	Hoxa5-/-	JJ031_S15	Single-end	42027412	42016977	38357572	91.29	68.8
Trachea E15.5	Hoxa5-/-	JJ032_S16	Single-end	35978554	35969080	32563604	90.53	67.2
Lung E15.5	wt	JJ033_S17	Single-end	58618931	58611081	52944871	90.33	69
Lung E15.5	wt	JJ034_S18	Single-end	52096679	52085799	47005759	90.25	68.3
Lung E15.5	wt	JJ035_S19	Single-end	41632020	41626847	37396881	89.84	68.6
Lung E15.5	wt	JJ036_S20	Single-end	38382017	38377126	34644058	90.27	68.5
Lung E15.5	Hoxa5-/-	JJ037_S21	Single-end	45918471	45908842	41386186	90.15	68.6
Lung E15.5	Hoxa5-/-	JJ038_S22	Single-end	55901388	55889032	50290253	89.98	68.9
Lung E15.5	Hoxa5-/-	JJ039_S23	Single-end	70463023	70455106	63294763	89.84	68.7
Lung E15.5	Hoxa5-/-	JJ040_S24	Single-end	32600711	32596229	27339550	83.87	54.8
Diaphragm E15.5	wt	JJ041_S25	Single-end	29774494	29768726	26940290	90.5	69.6
Diaphragm E15.5	wt	JJ042_S26	Single-end	39594445	39585429	35845217	90.55	69
Diaphragm E15.5	wt	JJ043_S27	Single-end	37441893	37436925	33674406	89.95	68.5
Diaphragm E15.5	wt	JJ044_S28	Single-end	47990655	47983941	43502940	90.66	69
Diaphragm E15.5	Hoxa5-/-	JJ045_S29	Single-end	47124763	47113526	42606554	90.43	69.1
Diaphragm E15.5	Hoxa5-/-	JJ046_S30	Single-end	64258184	64243936	58151267	90.52	68.8
Diaphragm E15.5	Hoxa5-/-	JJ047_S31	Single-end	45230556	45223132	40493430	89.54	67.3

Diaphragm E15.5	Hoxa5-/-	JJ048_S32	Single-end	57239273	57227131	51516527	90.02	68.7
Somites E10.5	wt	JM01	Paired-end	34342635	34335027	31560194	91.92	73.4
Somites E10.5	wt	JM02	Paired-end	34869522	34860473	32121412	92.14	75.3
Somites E10.5	wt	JM04	Paired-end	34324218	34315577	31814053	92.71	76.2
Somites E10.5	wt	JM05	Paired-end	30360985	30351849	28096591	92.57	73.8
Somites E10.5	Hoxa5-/-	JM06	Paired-end	33348397	33338468	31033586	93.09	76.7
Somites E10.5	Hoxa5-/-	JM07	Paired-end	33630019	33617068	31173526	92.73	77.8
Somites E10.5	Hoxa5-/-	JM08	Paired-end	32821852	32810961	30412285	92.69	77.7
Somites E12.5	wt	JM17	Paired-end	34928151	34916167	32577081	93.3	78.8
Somites E12.5	wt	JM18	Paired-end	34109162	34096128	31765548	93.16	79.4
Somites E12.5	wt	JM19	Paired-end	32515802	32505102	30317013	93.27	79.3
Somites E12.5	wt	JM20	Paired-end	33947543	33935982	31639478	93.23	78.8
Somites E12.5	Hoxa5-/-	JM21	Paired-end	32501825	32493670	30362829	93.44	78.1
Somites E12.5	Hoxa5-/-	JM22	Paired-end	33239446	33231294	31076333	93.52	78.4
Somites E12.5	Hoxa5-/-	JM23	Paired-end	30949813	30940950	28715124	92.81	79.4
Somites E12.5	Hoxa5-/-	JM24	Paired-end	31133030	31125300	29040532	93.3	79.3
iBAT E18.5	wt	319.2.WT	Paired-end	29130581	29121237	23917225	82.13	69.5
iBAT E18.5	wt	321.5.WT	Paired-end	27954956	27944515	22601560	80.88	68.7
iBAT E18.5	wt	324.5.WT	Paired-end	27524929	27513795	22414555	81.47	68.7
iBAT E18.5	wt	327.2.WT	Paired-end	24928936	24919844	20488516	82.22	70.6
iBAT E18.5	wt	327.4.WT	Paired-end	25501267	25490516	20979856	82.3	69.8
iBAT E18.5	Hoxa5-/-	319.1.Null	Paired-end	27684840	27675318	22641191	81.81	69.6
iBAT E18.5	Hoxa5-/-	321.1.Null	Paired-end	29596167	29587332	24249411	81.96	70
iBAT E18.5	Hoxa5-/-	324.1.Null	Paired-end	30791655	30777863	24894889	80.89	68.1
iBAT E18.5	Hoxa5-/-	327.1.Null	Paired-end	31271267	31258076	25391636	81.23	68.2
iBAT E18.5	Hoxa5-/-	327.5.Null	Paired-end	27244874	27232310	22007927	80.82	67.6

Supplementary Table 2. List of primer sequences for RT-qPCR assays

Gene	Sequence (5' - 3')	Fragment size (bp)
Hoxa1	F-AACTCCTTATCCCCTCTCCAC	151
	R-GACCCACGTAGCCGTACTC	
Hoxa2	F-AACAATGACAGTCCCGAGGC	98
	R-GGCGACAGTGCATCTGAAAG	
Hoxa3	F-GACCAGAAGGGCAAAGGCAT	158
	R-GGAGGCTTGGAGAAAGGAGG	
Hoxa4	F-GGTGGTGTACCCCTGGATG	82
	R-CGAGAGCGCTTAGGTTCGC	
Hoxa5	F-GCAAGCTGCACATTAGTCAC	78
	R-TCTGGTAGCGAGTGTAGGC	
Hoxa6	F-GGACTACCTGCACTTTTCTCCC	162
	R-CCGTGACTCCCATACACG	
Hoxa7	F-TTCTTGCTCCTTTGCACCCA	182
	R-TGTTTTGGTCGTAGGAGGCG	
Hoxa9	F-TGACTGTCCCACGCTTGACAC	185
	R-AGCATGTAGCCAGTTGGCAG	
Hoxa10	F-ACAGTAAAGCTTCGCCGGA	79
	R-CTCTTTGCTGTGAGCCAGTTG	
Hoxa11	F-CAGCCCCGAGTCGTCTTC	86
	R-TATAAGGGCAGCGCTTTTTGC	
Hoxa13	F-GTACTGCCCCAAAGAGCAGA	79
	R-GTCTGAAGGATGGGAGACGA	
Hoxb1	F-ACAACCCTTTTCCTGGCACA	180
	R-CCCCTTCTTGGTTGAGGCTT	
Hoxb2	F-CGTTTCATTCACTCCTTTTCGCT	119
	R-TTGTAAGAAAACCCTCTCCCACT	
Hoxb3	F-TGTCTGCCTGTTCTCCCCT	136
	R-TACCCTCACGACCGGACATT	
Hoxb4	F-AACCCCCTGCATCCCAGC	105
	R-GCCGGCGTAATTGGGGTTTA	
Hoxb5	F-AGCCCATGGCCACCTCTA	116
	R-CCGGGCCCTTTTTCCGT	
Hoxb6	F-GAGCGTGTTCGGAGAGACC	100
	R-GCTGGGCCCAAAAGAGGAAC	

Hoxb7	F-GCTCGAACCGAGTTCCTTCA<br>R-GCCTCGCTTTCGGTCAG	184
Hoxb8	F-ACTTCTACGGCTACGACCCT<br>R-CCTCGCCTGCGTCCG	196
Hoxb9	F-AAAGTGATGAGCCTCGCTCC<br>R-GCGTCTGGCTAGGTTACAGG	129
Hoxb13	F-GGAGGGGGTCGGAATCTAGT<br>R-GTTGACAGTTGGCATCAGCG	81
Hoxc4	F-GTAGATGCTCCCCCTACCCT<br>R-TGATCACGGGGCATTTCACA	116
Hoxc5	F-ATTTTGAGGCGGAGGCTAGA<br>R-CAGTACCCGCCCTACAAGAA	175
Hoxc6	F-TGTCGTGTTCAGTTCCAGCC<br>R-GGTCCGCTCCGTAACCG	242
Hoxc8	F-CATCTCCAACTCGGGCTACC<br>R-GCCTTGTCCTTCGCTACTGT	184
Hoxc9	F-CATCTGCTTTTGGCTGCACA<br>R-TGCGTTTTCCTGGCGATTTG	120
Hoxc10	F-TGCCCTCGCAATGTAACTCC<br>R-CTCATCACCCCGCAGTTGAA	128
Hoxc11	F-GCACCATCGGAACAGCTACT<br>R-TGTCGAAGAAGCGGTCGAAA	221
Hoxc12	F-ACTTCTGCTGAGGGAGCAAG<br>R-GCGTTGTAAGCGGGACTATG	127
Hoxc13	F-AGTCAGGTGTACTGCTCCAAG<br>R-CCGGTAGCTGCTCACTTCG	102
Hoxd1	F-AGACTGCTCCCCACCTTTGA<br>R-TGGCTCACATAGCAGCGTTTA	231
Hoxd3	F-CTAAGGGCATCCTGCATTCTCC<br>R-GTACATATTGGGCTGCGATTTGG	167
Hoxd4	F-GAAATGCACCGAGCCTACCT<br>R-CCCTCCTTACTCACCATCGC	116
Hoxd8	F-TCATTCTGGCAAGTGGCCTT<br>R-GTGGACGTACCAACCGACAT	195
Hoxd9	F-CCAAACAGGGCCAACGATCT<br>R-AGGTTTCTCGGTAGGCCTCT	166
Hoxd10	F-ACAGTTGGACAGACCCGAAC<br>R-CGGGGTTCTCAACAGGACAA	198
Hoxd11	F-ACACTCCAGGCAAACGAGAG	165

	R-GGCGGGCCAAGATTCCTTAT	
Hoxd12	F-CTCCGCCTTCCCCTACAATC	209
	R-AACACACCAACCGAGGTCAG	
Hoxd13	F-CACAGGGGTCCCATTTTTGGA	300
	R-AACCTGGCCCACATCAGGA	
Rpl19	F-GGCTTGCCTCTAGTGTCCTC	123
	R-TCAGCCCATCCTTGATCAGC	

Supplementary Table 3. List of primer sequences for ChIP-qPCR assays

# region	Sequence (5' - 3')	Fragment size (bp)
1	F-TTTGGTCCCCAGCACATTCA<br>R-GCCAGCCTCGGAATGTAGAG	76
2	F-ATGTCTCATTGTGGGACTCGG<br>R-GGACCATGAGCTTCGAGGGA	124
3	F-CAGGCAGGTCGATGGTACTC<br>R-GCGACCTACTACGACAGCTC	143
4	F-CTGTGGCGTCCCTTTGTAGA<br>R-GCTGGCTAGGAAGGAAGTCTG	151
5	F-CAGTTTTCTTCACCTGCCGC<br>R-GCATTTGGGAGGTGTCTGGA	88
6	F-GAGGGAACTTGGGCTCGATG<br>R-GAAAAACGACACCGCGAGAAA	104
7	F-GATGCCTCACTCGTACCTGG<br>R-TGGCGGTGATAGTTTGCAC	103
8	F-GCCCTGAAATGGAGCAGAGA<br>R-AATAGTGCTTCAACCCCGCT	82
9	F-ACCACCGCCTGATGGAATAG<br>R-AGGCTGGTTTGACAGGGAAC	79
10	F-GGGAAACCCTAACAAACCCCTA<br>R-AAAAGGAGAAGGTGGCAGAAGT	121
11	F-GCCCTGCGAAGACATATTGC<br>R-CTCTGGGAGTTGAAGGGACG	140
12	F-GCTTTGTTAGCCACACTCCG<br>R-AGTGCTGGGGTTGGTTAGTG	118
13	F-GCAACGAGAACAGGGCTTCT<br>R-GGGCAGCTCTCTGTAGTGTC	86
14	F-CAGGGTCTGGTAGCGAGTGT<br>R-GCTGAGGAGTTGGTGGCTAA	104
15	F-GGGTAGGGATGGCAGTAAGG<br>R-CAGCGTAGCCCTTCCCATAAA	71
16	F-CCCTCTCTGCTGCTGATGTG<br>R-GGAAAAACTCCCTGGGCAAC	73
17	F-CCAGTCGTAAATCCTGCCTGA<br>R-CTCTTGCCTCCACCCAACTC	75

18	F-TGCAGGACTGTGATTTGTTGTG	87
	R-CGTTCGGCCATCCAGAAAC	
19	F-GGAAGGGCCGAACAACTCA	131
	R-GACTCGGTGGTAAGATGGCG	
20	F-AGAGGGGGCTGTTGACATTG	112
	R-GAGCCGACTTCTTGCTCCTT	
21	F-CATCCTTAGCAGGGAGGTGC	171
	R-TGACGATCTGTTGCTTCCCC	
Rpl19	F-TGAAGCGATGGAGAGATGCG	71
	R-0CCCAGACCCTTGGAGTGTTC	
Olig2	F-GTAGGGTCTCCTCACACGGA	70
	R-CACAAGCAAGCGGAACCAAT	
